# FullThrOTTLE-TrIR: Time-Resolved IR Spectroscopy of Electrochemically Generated Species Using a Full Throughput Optically Transparent Thin-Layer Electrochemical Cell

**DOI:** 10.1021/acs.jpcc.4c04947

**Published:** 2024-09-18

**Authors:** Kerstin T. Oppelt, Peter Hamm

**Affiliations:** Department of Chemistry, University of Zurich, Zurich 8057, Switzerland

## Abstract

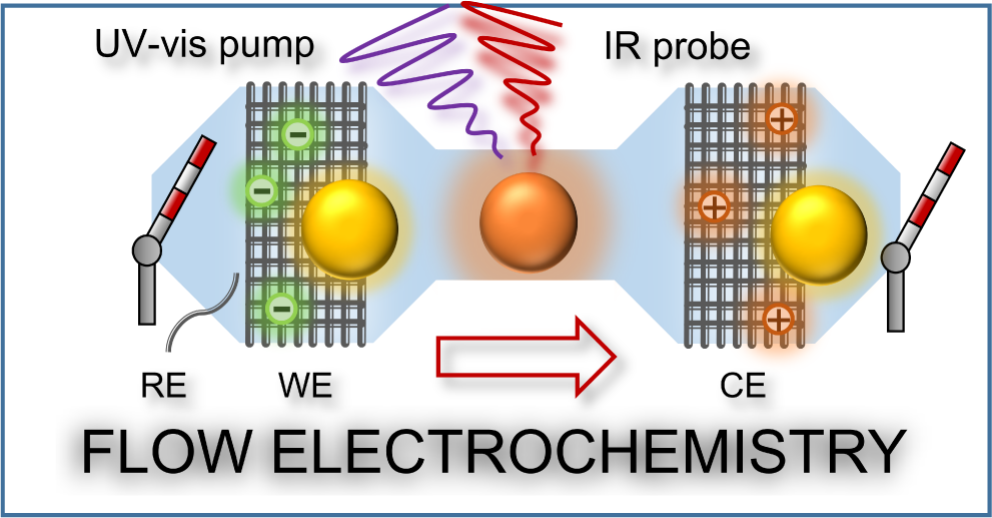

An optically transparent thin-layer electrochemical cell with stopped-flow sample transport has been developed for optical-pump infrared-probe transient absorption spectroscopy of prereduced or preoxidized molecules. Time-resolved IR-spectra of Re(bpy)(CO)_3_X (X = Cl, Br) complexes in different oxidation states are presented as a proof-of-principle application for this combined electrochemical and spectroscopic tool. The excited-state lifetimes and IR-spectroscopic signatures of various oxidation states of the molecule, including follow-up reaction intermediates, are disentangled by kinetic sorting, using lifetime density analysis. The method can be applied to assign and differentiate molecular intermediates in photo- and electrochemical reactions, adding new analytic coordinates to classical FTIR- and UV–vis-spectroelectrochemistry.

## Introduction

Photoredox catalysis is a central aspect of photochemical energy storage. Mechanistic electrochemical studies are key requirements for understanding the charge transfer processes involved and for the identification of reactive species. Spectroelectrochemistry (SEC) merges electrochemistry and spectroscopy and facilitates a comprehensive examination of both single and multiple electron-transfer processes and related chemical steps in photo- and electrocatalytic processes.^1^ UV–vis–NIR and IR absorption spectroscopy, NMR, and electron paramagnetic resonance (EPR), along with many other techniques, have been coupled to electrochemistry, some of them in combination.^2^

FTIR-SEC,^1,3−7^ especially using optically transparent thin layer electrochemical (OTTLE) cells,^8^ has evolved to be an indispensable part of catalytic mechanistic investigations of carbonyl compounds. These can be especially well observed in the IR, not only because of the large transition dipole moment of carbonyl modes but also due to the fact that these modes are sensitive to changes in the redox potential of the metal center and other ligands through back-bonding. The OTTLE cell design allows for measurements to be conducted in transmission mode with a small amount of sample. They enable the direct and real-time observation of molecules undergoing electrochemical transformations in solution and detailed studies of charge transfer and ligand exchange processes.^8^ FTIR-studies in an OTTLE cell are often accompanied by separate transient-IR (trIR) kinetic measurements to gain mechanistic information on faster time scales. To address the fact that one can typically trigger these reactions starting from only a stable oxidation state and the fact that observation is often limited to the first electron transfer step, we propose here to combine electrochemistry and trIR-spectroscopy into a single method.

Light-induced FTIR difference experiments have been performed in thin-layer transmission cells^9^ and ATR geometry.^10^ Electrochemistry has also already been combined with time-resolved infrared spectroscopy (IR pump–IR probe) in three main geometries: reflection of the probe beam by the working electrode,^11−13^ internal reflection on an ATR-prism,^14^ and transmission through a grid^15^ or semitransparent working electrode.^16^ Each have specific advantages and disadvantages but are aided by the fact that vibrational excited states of molecules in solution are typically very short-lived, <100 ps, with exceptions that reach 1–2 ns.^17^ For similarly short-lived processes, such as the excited-state relaxation in our demonstration example, it is possible to use similar setups for pump–probe spectroscopy with electronic excitation.

In contrast, for electronic excitation that induces permanent changes, as is typically the case for electrochemical reactions, sample exchange is required, as soon as the phototriggered processes last longer than the time between consecutive pump laser shots. There are different approaches to doing that: pumping the sample solution through the overlap of pump and probe beam^18,19^ or rotating and/or rasterizing the static sample.^20−22^ In combination with electrochemistry, we have to find a compromise between efficient sample electrolysis and exchange. For example, 2D electronic electrochemical spectroscopy has been realized by pre-electrolyzing a large volume of sample and then measuring time-resolved electronic spectroscopy outside of the electrochemical system in a conventional flow cell.^23^ This was possible because the sample possessed stable (and reversible) redox states. This approach should also work in combination with time-resolved vibrational spectroscopy; however, here we present a different route.

Our approach to sample exchange is by (stopped-)flow through an electrochemical flow cell, which we coin “fullthroughput optically transparent thin-layer electrochemical cell” (FullThrOTTLE cell). When the sample is pumped through the cell, it is electrochemically altered at both the working and counter electrodes, while the time-resolved measurement happens between these electrodes. We will use the sample flow rate as an additional handle in this throughput electrolysis cell. By adjusting the residence time at the electrode and thus the extent of reduction/oxidation experienced by the system, intermediates can be selectively targeted depending on their stability. To that end, we modified the standard OTTLE cell^8^ to a flow cell, which allows us to control the degree of reduction/oxidation by adjusting the flow rate through the cell. Additionally, we included the possibility of synchronizing the sample exchange with the laser measurement for trIR-measurements of relatively slow processes.

## Methods

### FullThrOTTLE Cell Design and Characterization

**Figure 1:**
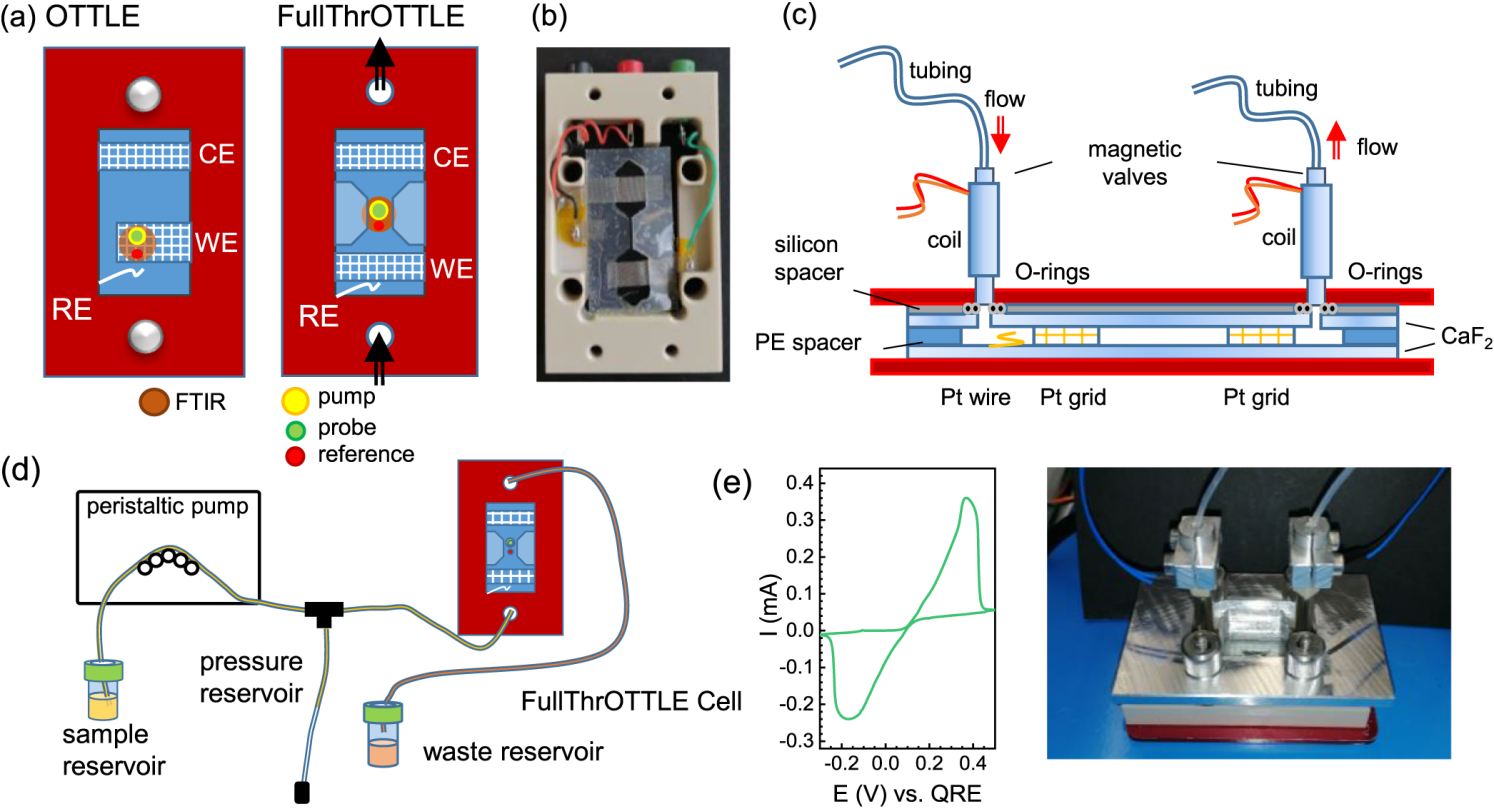
(a) Cell design for a classic optically transparent thin-layer electrochemical cell (OTTLE cell^8^) versus an optically transparent thin-layer electrochemical cell with flow configuration (FullThrOTTLE cell). (b) Picture of the actual spacer as mounted in the spacer holder. (c) Cut through the middle of the cell to visualize how the valves and coils are mounted. (d) Illustration of the FullThrOTTLE cell implementation for trIR measurement. (e) Thin-layer cyclic voltammogram (TL-CV) of the Fc/Fc^+^ redox pair in the FullThrOTTLE cell. (f) Picture of the cell assembly as shown in panel (c).

Figure 1a shows a schematic comparison between a conventional OTTLE cell and a FullThrOTTLE cell design. In an OTTLE cell, the detection beam goes through the working electrode and the sample is static. In contrast, in the FullThrOTTLE cell, the sample is pumped through the cell (either continuously or in a pulsed mode); it is reduced or oxidized at the working electrode, and the measurements are done immediately downstream in the narrow measurement channel. We regulate the sample flow through the cell by two controls: first, the pressure produced by the peristaltic pump, and second, by magnetic valves whose opening intervals and times can be electronically controlled.^19^Figure 1f shows a picture of the mounted cell, and Figure 1b shows the spacer design. The much larger channel width at the working electrode leads to slow flow at the electrode and efficient reduction and fast sample exchange in the measurement channel. Figure 1c shows a cut through the cell, the connection to the magnetic valves, and the spacer and the O-rings necessary for the connection of the CaF_2_ windows with holes. Figure 1d shows the overall flow system; a more detailed description can be found in the Supporting Information. The sample can be measured only once, as the reaction at the counter electrode at the top of the cell is undefined and can permanently alter the sample.

Electrochemical testing of the FullThrOTTLE cell was done by cyclic voltammetry of ferrocene; see Figure 1e. The large peak separation of more than 500 mV upon scanning a CV in the FullThrOTTLE cell at 2 mV s^–1^ shows that there is significant transport resistance within the cell. The Fc/Fc^+^ half-wave potential was around 0.1 V vs a quasi-reference electrode (QRE). We will use the potential values applied versus the QRE for all measurements performed under flow, but need to keep in mind that the value vs Fc/Fc^+^ should be roughly 0.1 V more negative, based on the results of Figure 1e.

### Sample Preparation

The synthesis of ReCl and ReBr was conducted by following established protocols^24^ by refluxing Re(CO)_5_X (where X = Cl, Br) in deoxygenated toluene for 5 h. After cooling the resulting orange solutions in a refrigerator, a yellow precipitate was filtered off using a Buchner funnel and washed with small amounts of ice-cold toluene. The solid was dried on the HV.

For all spectroscopic and electrochemical measurements, either tetrabutylammonium hexafluorophosphate (TBAPF_6_) or tetrabutylammonium chloride (TBACl) was used as the electrolyte in dry acetonitrile as the solvent. The sample concentration was 5 mM, and the electrolyte concentration was 100 mM unless otherwise stated.

### UV–vis and FTIR SEC

Parallel UV–vis and FTIR spectroelectrochemistry was performed using a Bruker Tensor 70 spectrometer and a UV–vis Ocean Optics fiber spectrometer. A standard steady-state OTTLE cell was used for the CV mode SEC experiments at 1 mV s^–1^ with an Ivium potentiostat. Spectra were recorded at defined time intervals of 5 s for UV–vis (integration time was adjusted to optimize the probe light level but was about 20 ms) and 15 s for FTIR with 4–8 scans of the interferometer at a resolution of 2 cm^–1^. The FTIR acquisition was sometimes less frequent but in sync (±1 s) with the UV–vis absorption.

FTIR data were background corrected with the FTIR spectrum of acetonitrile in the OTTLE cell. The first reductive wave in the simultaneously acquired CV was referenced against the first reduction wave from classical CV based on the reported value of −1.77 V vs Fc/Fc^+^.^7^ Subsequently, the derivative in the voltage direction was calculated. In the case of UV–vis-SEC data, this was done after applying an SVD filter to reduce noise.

### Time-Resolved Infrared Spectroscopy (TrIR)

The measurement of the trIR pump–probe spectra was performed above the working electrode in the measurement channel of the FullThrOTTLE cell (see Figure 1), using either tetrabutylammonium hexafluorophosphate (TBAPF_6_) or tetrabutylammonium chloride (TBACl) as the electrolyte in dry acetonitrile as the solvent. We conducted measurements of spectra of ReCl (Re(bpy)(CO)_3_Cl) and (ReBr Re(bpy)(CO)_3_Br) at different working electrode potentials and flow rates. This way, the sample residence time at the working electrode was varied. This established selected mixtures of redox intermediate species in the interaction region (where the laser measurement was performed). Observation of the electrolysis current fluctuations helped to estimate how stable the flow in the cell was. The valve opening frequency and opening time can be adjusted to control the residence time of the sample at the working electrode (WE). The prepressure in the reservoir, however, has the greatest influence on the flow behavior in the cell. The most practical approach was to keep the valve opening time constant (at 750 μs) and the prepressure as constant as possible by adjusting the rpm value of the peristaltic pump and the pressure applied on the flexible tubing over the pump head rollers. It is then easy to only adjust the opening rate while observing the pump–probe signal after *t*_0_ to obtain the desired starting conditions and then adapt the peristaltic pump speed to keep the prepressure constant (i.e., the level of liquid in the pressure reservoir). It will vary due to the spacing of the rollers on the peristaltic pump head (see Figure 1d), as there is always a pressure drop at the interchange of two rolls. Increasing pressure buildup in the pressure reservoir during the measurement has to be avoided as this greatly influences the flow and, as such, the sample composition. A good observable to check for consistency is the observation of a stable current in the chronoamperometry and stability of the initial trIR signal at *t*_0_. Giving the system 2–5 min before starting the trIR data acquisition to confirm this turned out to be a feasible approach. Due to the stop-flow functionality and small spot size of the laser, sample consumption is very small, so not much sample is lost during that time. Samples are used up in the measurement as soon as a potential is applied: after passing the measurement channel, it passes the counter electrode where the reactions happening to counterbalance the charges are undefined and most likely irreversible.

Samples for the trIR measurements were purged with Ar. Time-resolved IR spectra covering time scales from 10 ps–40 μs were obtained using a system of two electronically synchronized 2.5 kHz Ti-Sapphire laser systems.^25^ The first laser system is used to produce the IR probe light. These pulses are obtained via optical parametric amplification followed by difference-frequency mixing of the 780 nm fundamental beam.^26^ 420 nm pump pulses were generated by frequency doubling of 840 nm light from a second Ti-Sapphire laser. For measurements with an 840 nm pump pulse, the fundamental of the Ti-Sapphire laser was attenuated appropriately. Since the changes in the trIR spectra became very broad in terms of wavelength ranges, the full spectral range of the OPA output was used in selected cases. Two different positions of the spectrograph gratings were alternated for each time delay in these cases.

### Data Analysis of TrIR Measurements

Data sets that were recorded with more than one center wavelength setting of the spectrograph grating (100 lines/cm) in order to cover a larger spectral range were combined, and their frequency scale was corrected using a linear function based on a comparison of the bleach positions in the trIR (with open valves and without applied potential) with FTIR spectra of the parent molecules. All combined data sets were interpolated with a higher resolution of 2 cm^–1^ to avoid spectral distortions from the original resolution of about 4–6 cm^–1^.

**Figure 2:**
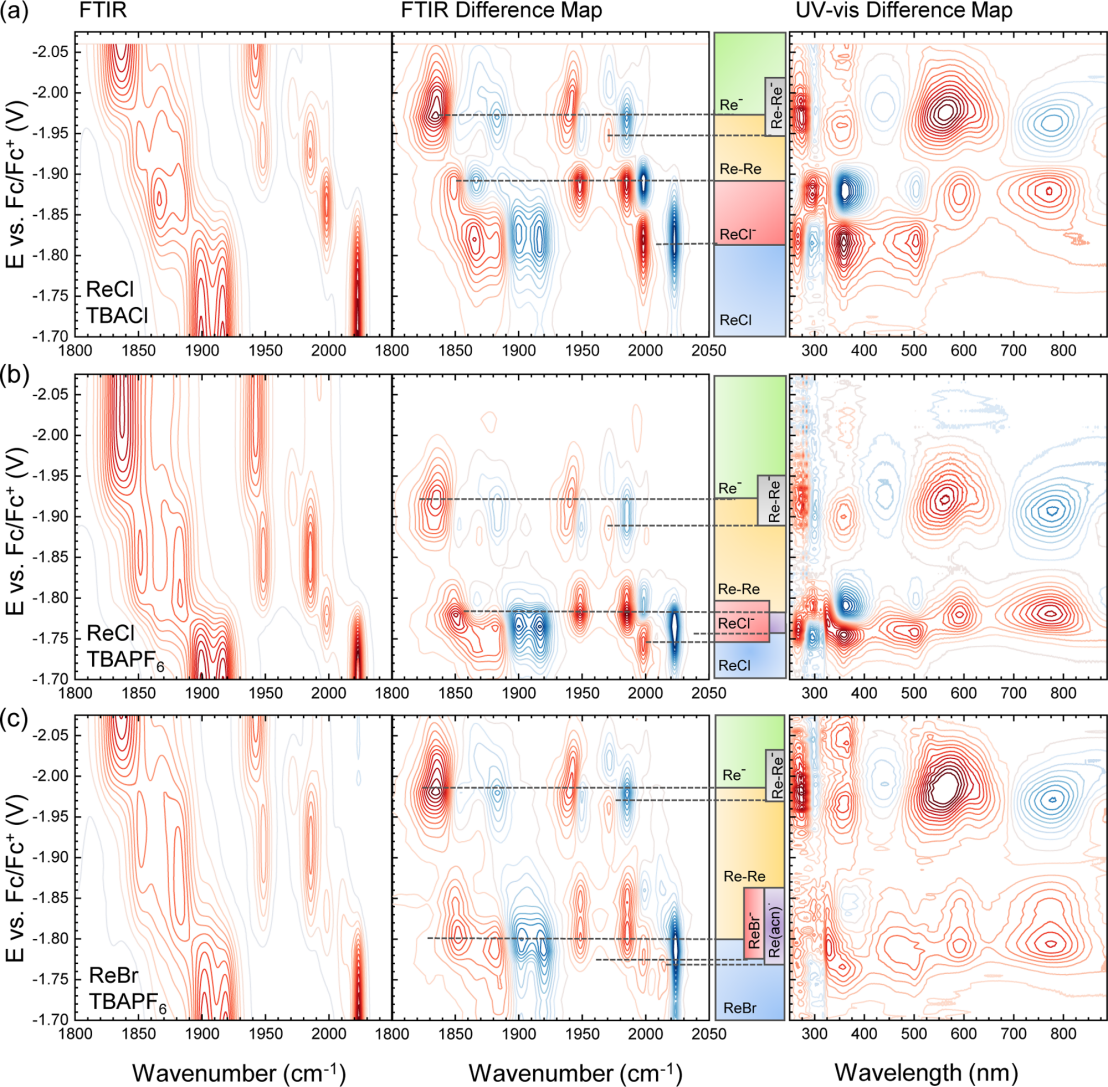
Standard SEC of (a) ReCl in TBAPF_6_, (b) ReCl in TBAPF_6_, and (c) ReBr in TBAPF_6_ measured in a classic steady-tstate OTTLE cell at 1 mV s^–1^ in the FTIR and UV–vis range. Positive contours are depicted in red, and negative signals are depicted in blue. In the left column, FTIR-SEC spectra are shown; in the middle and right columns, the difference maps of the FTIR and UV–vis SEC are plotted. The colored bar graphs between the difference maps indicate the voltage range where various species can be observed. The start of each bar was set to the maximum of the respective positive signal (red) in the IR difference map (middle), as indicated by dashed lines. The end of each bar is at the minimum of the negative peak (blue) in the FTIR-SEC difference map.

**Figure 3:**
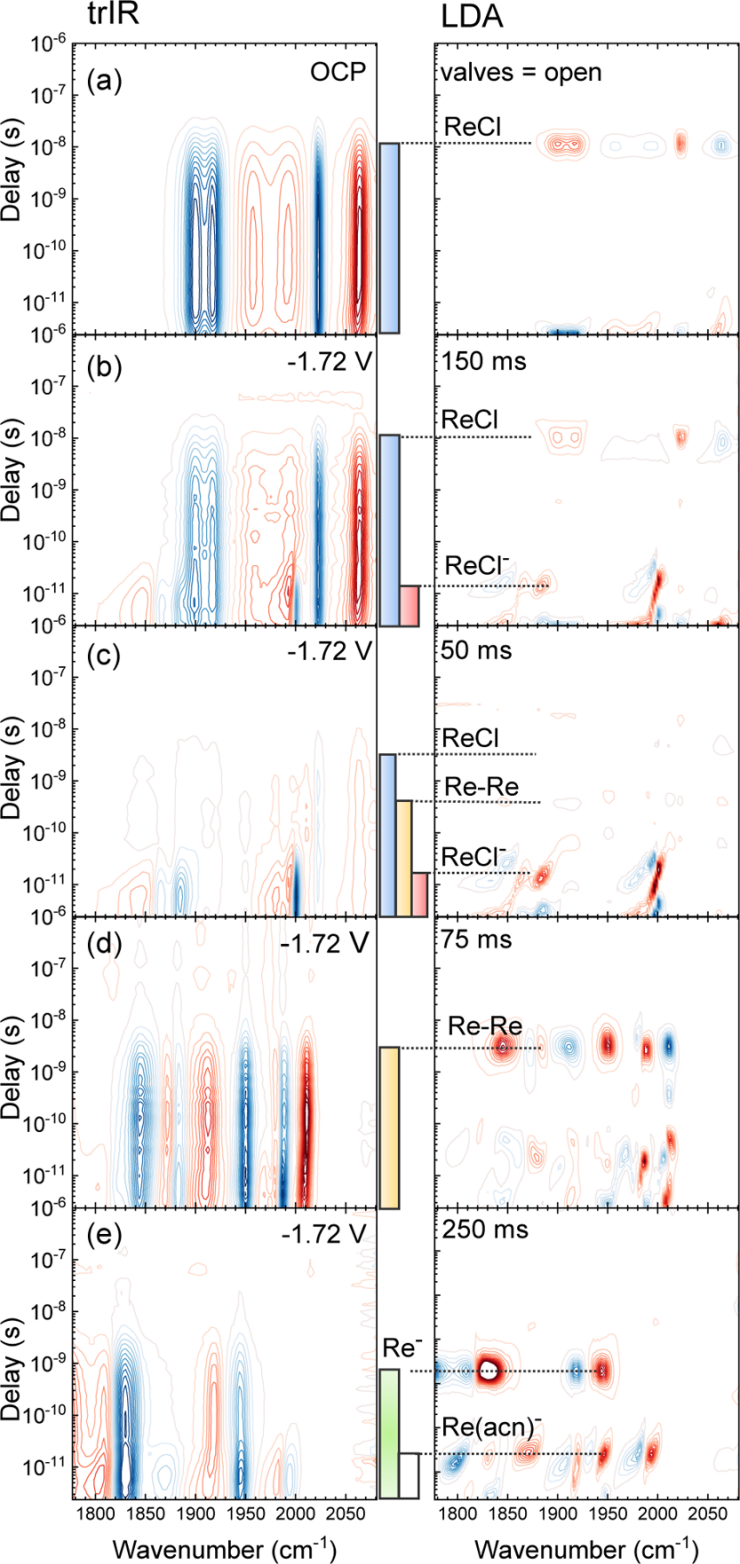
FullThrOTTLE-trIR results of ReCl with TBACl in acetonitrile with an increasing residence time at the working electrode from panel (a) to panel (e). The left column shows the trIR data and the right column shows the corresponding lifetime analysis. The color bars in the middle indicate the excited-state lifetimes of the different species, using the same color code as in Figure 2. Valve opening intervals are given at the top of each panel. The data in (b) were measured at a higher prepressure, hence the large opening interval compared to the following data sets.

**Figure 4:**
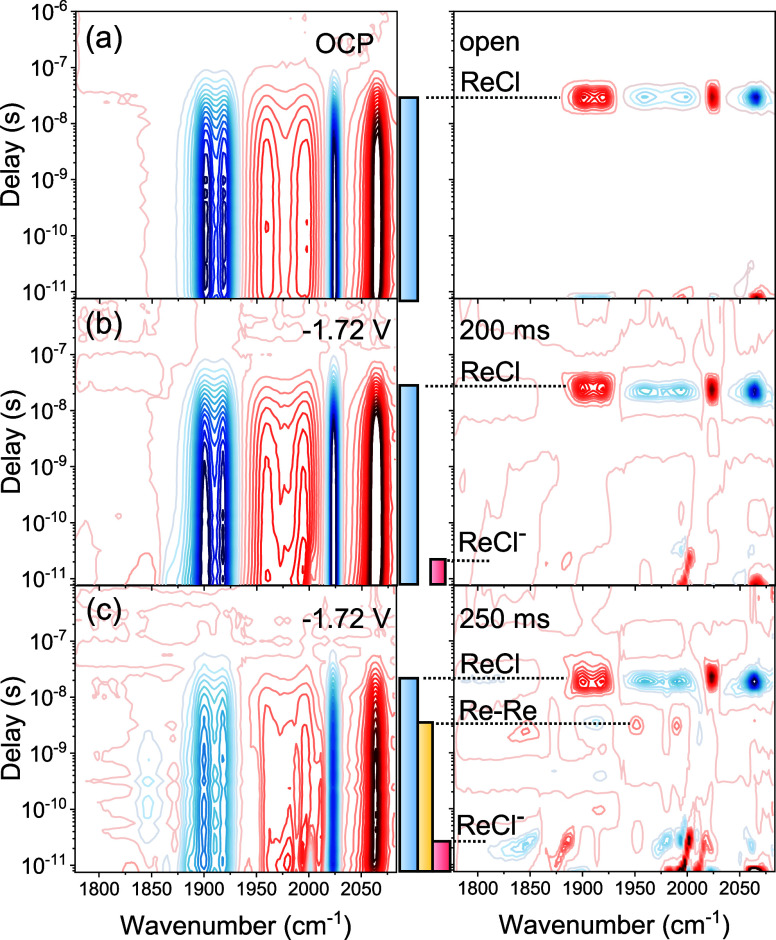
FullThrOTTLE-trIR of ReCl with TBAPF_6_ in acetonitrile under different flow conditions, increasing the degree of reduction from panel (a) to panel (c). The left column shows the measured spectra, and the right column contains the corresponding lifetime density maps as obtained by lifetime density analysis. The color bars in the middle indicate the excited-state lifetimes of the different species, using the same color code as in Scheme 1 and Figure 2.

**Figure 5:**
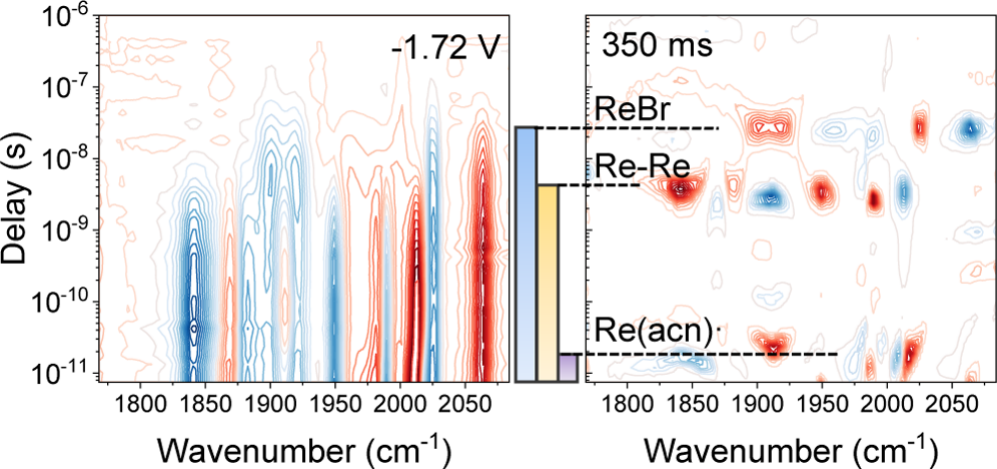
FullThrOTTLE-trIR results of ReBr with TBAPF_6_ in acetonitrile. The left column shows the trIR data, and the right column shows the corresponding lifetime analysis. The color bars in the middle indicate the excited-state lifetime of the different species, using the same color code as that in Scheme 1.

**Figure 6:**
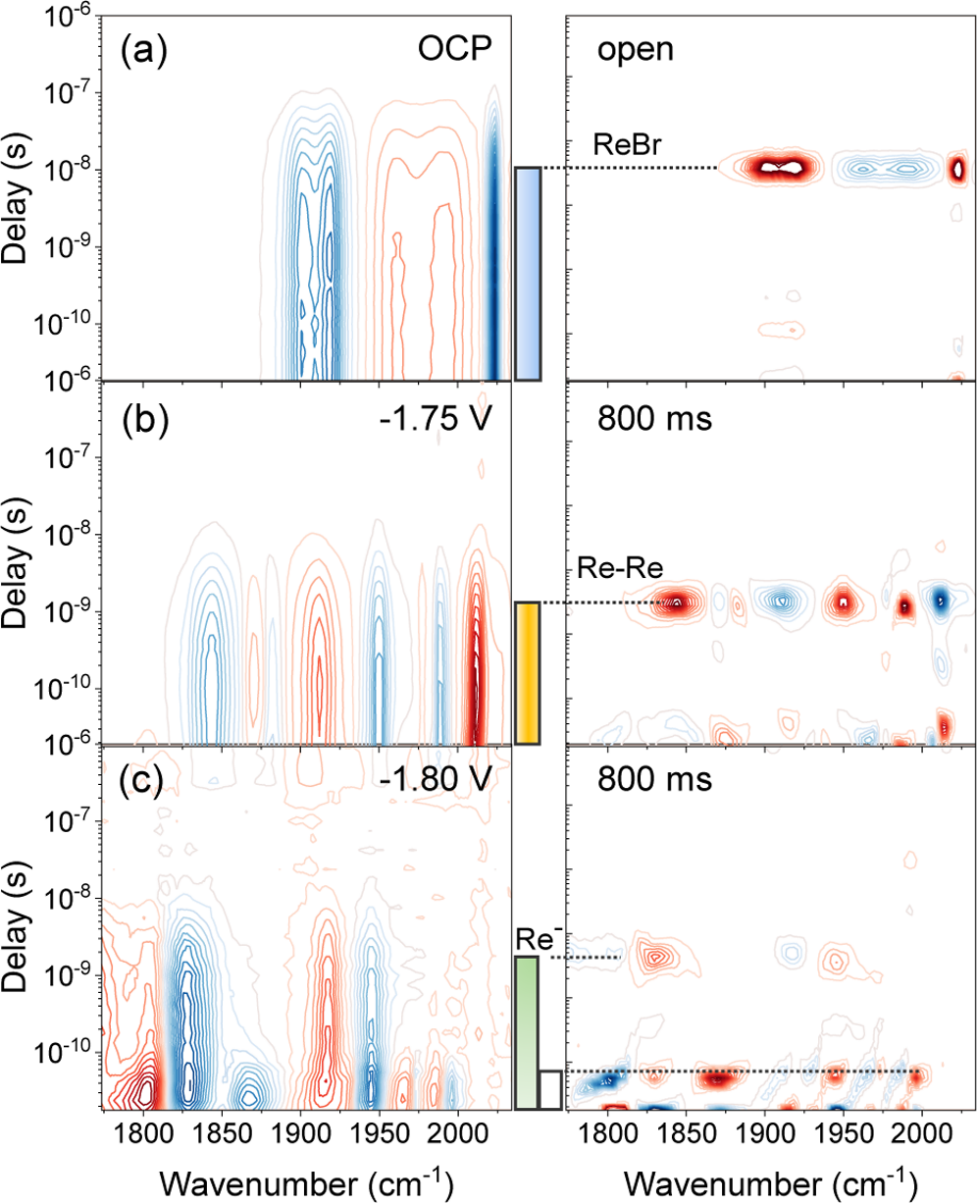
FullThrOTTLE-trIR of ReBr with TBAPF_6_ in acetonitrile. (a) Without the applied potential, (b) at −1.75 V, and (c) at −1.8 V vs QRE at very long valve opening intervals (800 ms). The left column shows the trIR data, and the right column shows the corresponding lifetime analysis. The color bars in the middle indicate the excited-state lifetime of the different species, using the same color code as in Scheme 1.

**Figure 7:**
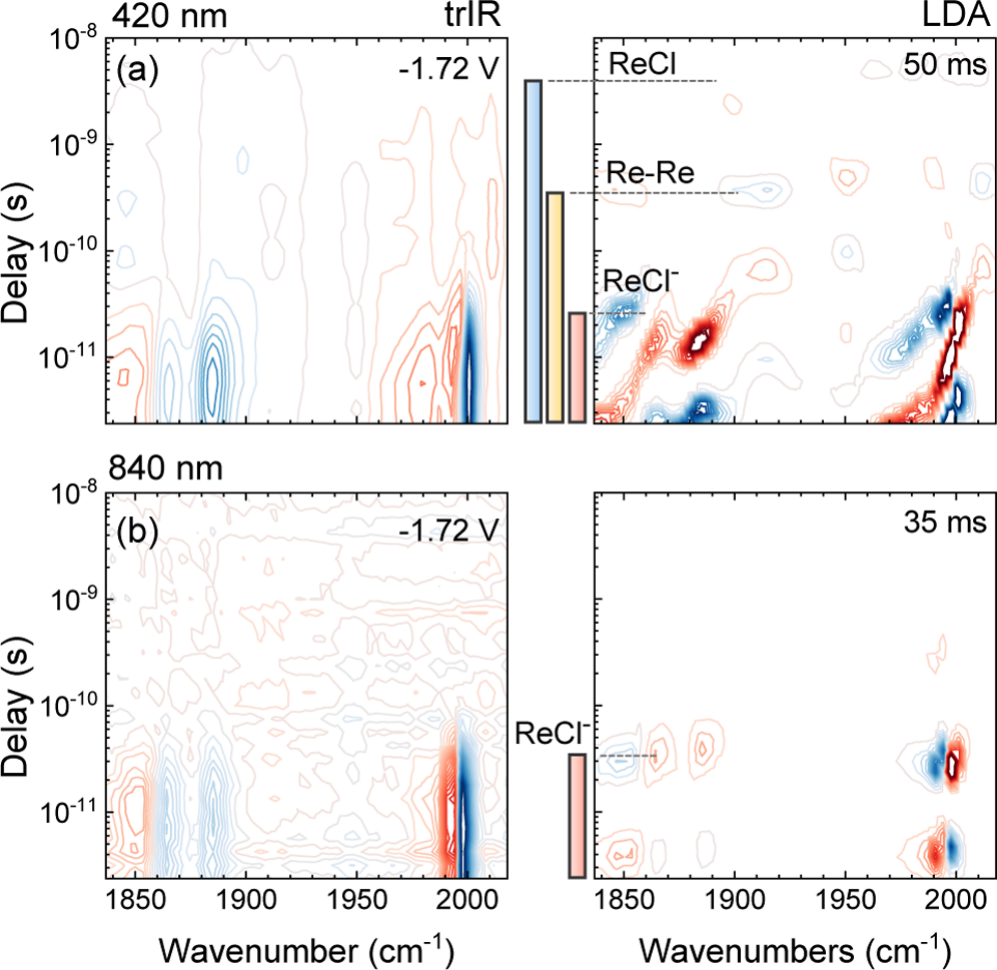
FullThrOTTLE-trIR of ReCl with TBACl in acetonitrile excited at (a) 420 and (b) 840 nm. The left column shows the trIR data, and the right column shows the corresponding lifetime analysis. The color bars in the middle indicate the excited-state lifetime of the different species, using the same color code as in Scheme 1.

The trIR-data were analyzed by lifetime analysis (right columns in Figures 3–7), which to a certain extent are analogous to the difference maps in Figure 2, but here spectral change occurs with respect to pump–probe delay on a logarithmic scale. To that end, the kinetic traces at each frequency ω_*i*_ were fitted to a sum of exponential functions with time constants τ_*k*_:
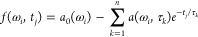
1The time constants τ_*k*_ are fixed and distributed equidistantly on a logarithmic scale with 10 terms per decade, and the amplitudes *a*(ω_*i*_, τ_*k*_) are the free fitting parameters. A maximum entropy method was applied for the fit, the procedure would yield very unstable results otherwise.^27,28^ This type of lifetime analysis produces lifetime density maps instead of discrete time constants. Maxima in this map indicate areas of maximum signal change (maximum slope) at a certain time. This visualizes at which delay the spectrum changes and shows which bands likely belong to one-and-the-same species, as these signals should change simultaneously. The combination of different flow conditions and lifetime density maps can thus help to disentangle otherwise quite congested spectra.

## Results and Discussion

**Scheme 1:**
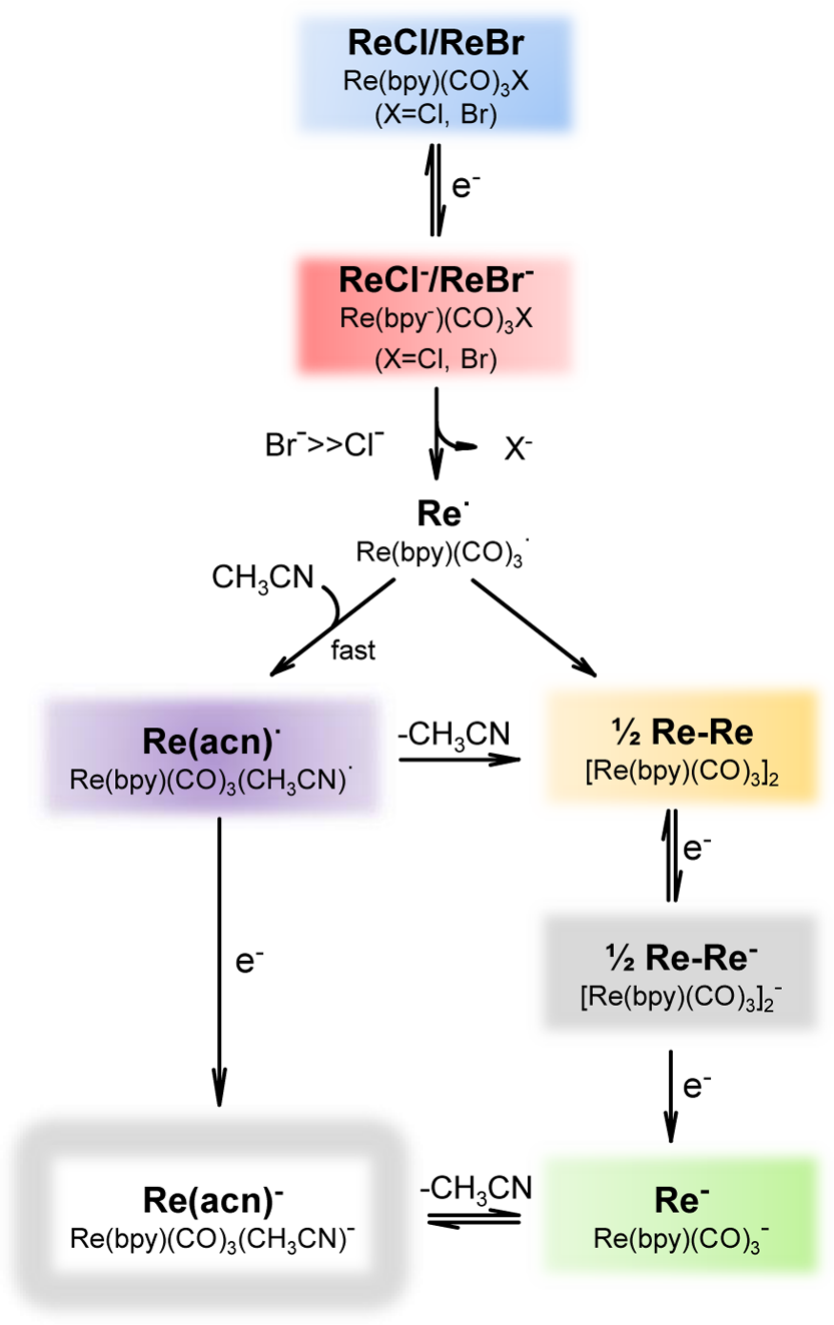
Reaction Intermediates in the Reduction of Re(bpy)(CO)_3_X (X = Cl, Br) as Described in the Literature^4,5,29,30^ The abbreviations (bold) are used throughout the following description, as is the color code used.

To demonstrate the capability of the FullThrOTTLE cell, we used Re(bpy)(CO)_3_X with X = Cl (ReCl) and X = Br (ReBr) as samples dissolved in acetonitrile with tetrabutylammonium chloride (TBACl) or tetrabutylammonium hexafluorophosphate (TBAPF_6_) as conducting electrolytes. The three combinations – ReCl with TBACl, ReCl with TBAPF_6_, and ReBr with TBAPF_6_ – exhibit different stability of the Re–X bond of the singly reduced complexes.^4^ Different intermediate species following the reduction of ReCl and ReBr have been discussed in the literature.^4,5,29,30^Scheme 1 summarizes the reduction intermediates of ReCl and ReBr and should serve as a reference for the naming and color coding we used to label the species we observed throughout this work.

In the following, we first describe the standard SEC of these samples in CV mode, the setup for the FullThrOTTLE-trIR method, and the additional insights into the reactivity in these systems we obtained from the FullThrOTTLE-trIR experiments. We will also demonstrate the combination of flow-controlled electrolysis with lifetime density analysis as an analytic tool and discuss the specific advantages and limitations of the FullThrOTTLE approach.

### UV–vis and FTIR Spectroelectrochemistry in the OTTLE Cell

The left column of Figure 2 shows contour plots of the C≡O stretch absorption as a function of applied potential for the three sample/electrolyte combinations measured with a conventional OTTLE cell. The whole sample has been reduced at the working electrode in these experiments. We cover the range from −1.7 V vs Fc/Fc^+^ (i.e., before the first reduction step) to −2.07 V (after the second reduction step). The Re–X bond becomes less stable when going from the system in Figure 2a with ReCl in a Cl^–^-containing electrolyte to the system in Figure 2c with ReBr in TBAPF_6_. In either case, the three typical ν_CO_ tricarbonyl vibrational modes (A’(1), A”, and A’(2))^31^ are observed at ≈2020, 1915, and 1900 cm^–1^, respectively, at the starting potential of −1.7 V. With the increasing negative potential, they shift to lower frequencies in steps corresponding to reduction or subsequent chemical reactions. Ranges where different species can be observed are indicated by colored bars between the middle and right columns of Figure 2. The boundaries of these colored bars are determined from the maxima in the FTIR-difference map (derivative of the FTIR spectra with respect to the applied potential) in the middle column of the figure. They align with the corresponding difference map of the UV–vis absorption in the right column of Figure 2, which, however, has lower resolution than the IR spectra.

We start by discussing the simplest example, i.e., Re(bpy)(CO)_3_Cl in TBACl, as shown in Figure 2a. The parent compound is marked with a blue color bar and exists down to −1.81 V. The red bar marks the stability range of the singly reduced species Re(bpy^•–^)(CO)_3_Cl (ReCl^–^) (bands at 1998, 1882.9, and 1866.2 cm^–1^), which appear at −1.81 V and disappear again at −1.89 V. In the corresponding UV–vis spectrum, the largest changes happen around 350 and 505 nm.

The stability region of the next set of signals is marked with an orange bar: two CO bands in the higher energy region (1985.1 and 1947.8 cm^–1^) are well separated. The parallel changes in the 1800–1950 cm^–1^ region are not well resolved in the contour plot but can be seen in the corresponding difference map (middle column) at 1881.6 and 1850.8 cm^–1^. All four IR-bands appear simultaneously in CV-SEC and are associated with new absorption bands in UV–vis at 590 and 770 nm. The signals of the singly reduced Re(bpy^•–^)(CO)_3_Cl complex decrease in parallel. In the past, carbonyl signals at these positions have been assigned to an equilibrium between two species: [Re(bpy)(CO)_3_]^−^ (Re^–^) and [Re(bpy)(CO)_3_(acn)]^−^ (Re(acn)^−^).^5^ A more recent study has instead assigned all four bands to a single species, i.e., the Re–Re dimer [[Re(bpy)(CO)_3_]_2_] (Re–Re), based on 2D-IR spectroscopy that can determine the connectivity between modes.^15^

At potentials <2.1 V, the final reduction product with a narrow feature at 1947.7 cm^–1^ and a broader feature at 1836 cm^–1^ is observed, which we assign to [Re(bpy)(CO)_3_]^−^ and mark with a green bar. The visible absorbance shifts back to 560 nm upon that transition.

Intermittently, between −2.05 and −2.15 V, a pair of smaller signals at even lower energy (1971–1973 and 1870 cm^–1^) can be seen (marked in gray). By comparison with reported data in THF,^30^ this could be the one-electron reduced Re–Re dimer (Re–Re^–^). It is the only reported species that has an absorption in the 1974 cm^–1^ region and is formed after the observation of the presumed Re–Re dimer. In the UV–vis difference map, this intermediate (gray marker) has no clear signature.

Without the excess of Cl^–^ from the electrolyte, i.e., for ReCl in TBAPF_6_, the stability ranges change; see Figure 2b. The first signal for Re(bpy^•–^)(CO)_3_Cl (red marker) is observed over a much smaller voltage range, whereas the four parallel follow up modes (orange marker) persist longer. This confirms that Cl^–^ loss and thus follow up reactions are facilitated in the absence of excess Cl^–^ in the electrolyte. There is a tiny 2010 cm^–1^ signal, marked in violet, between −1.74 V and −1.76 V, indicating the formation of small amounts of [Re(bpy(CO)_3_(acn)]^•^ (Re(acn)^•^).^5^

For the ReBr/TBAPF_6_ system (Figure 2c), the halogenated radical anion Re(bpy^•–^)(CO)_3_Br (ReBr^–^) signal (at 1998.6 cm^–1^) is very small in the FTIR-SEC; however, it is observable over a similar range in the form of a shoulder at 2010.8 cm^–1^ next to the initial signal of ReBr. This species has been assigned to [Re(bpy(CO)_3_(acn)]^•^,^5^ reflecting the decreased stability of the Re–halogen bond in Re(bpy^•–^)(CO)_3_Br. The aforementioned set of four bands (orange label) has the largest voltage range from −1.82 V to −2.0 V in the ReBr/TBAPF_6_ system. The final signal beyond −2.15 V is the same for all samples. The small “transition feature” at 1872 cm^–1^ (gray label) is also visible.

### FullThrOTTLE-TrIR

Figure 3 shows the time-resolved IR-spectra of ReCl in TBACl under different flow conditions in the FullThrOTTLE cell. We chose a pump laser wavelength of 420 nm, where the UV–vis SEC difference map shows only small changes (see the right column in Figure 2a), and hence, we expect little selectivity for any of the reduced species in the trIR measurement. A constant potential of −1.72 V was applied (with the exception of Figure 3a); however, when comparing this value to Figure 2, one must keep in mind that it was measured vs a QRE and might be off by a few 100 mV from Fc/Fc^+^; see Figure 1e. The sample residence time at the working electrode was gradually increased from Figure 3a to e by increasing the opening intervals of the valves (the data in Figure 3b were measured at a higher prepressure, hence the large opening interval compared to the following data sets). Negative difference signals in the trIR signals, i.e., the ground-state bleach (GSB), are shown as blue contour lines, and positive signals such as the excited-state absorption (ESA) and reaction product signals are shown in red. The left column shows the actual trIR data, and the right column shows the result of a lifetime analysis (see the Methods section for details). The colored bars in between the two columns indicate the species present in the interaction region, using the same color code as in Scheme 1 and Figure 2.

Figure 3a shows the trIR spectrum of ReCl in the FullThrOTTLE cell without an applied potential (OCP). It exhibits the typical MLCT/L’LCT signature of ReCl*^32^ with a red shift of the vibrational modes upon excitation. ReCl* ESA bands are found at around 2060, 1958, and 1993 cm^–1^ for the A’(1), A”, and A’(2) modes of the tricarbonyl vibrations, respectively.^31^ The lifetime analysis shows peaks at 11–12 ns, revealing the triplet excited-state lifetime of ReCl*^33^ in TBACl/acetonitrile.

In Figure 3b, under fast flow conditions, this first set of bands can still be seen but is smaller. Additionally, there are new GSB signals of the singly reduced radical anion with the A’(1) mode around 2000 nm and the bleaches of the asymmetric modes at 1887 and 1867 cm^–1^. The corresponding ESA signals are all shifted to lower frequencies and partially overlap with the bleaches, leading to maxima at 1992 and 1945 cm^–1^. The frequency down-shift of the ReCl^–^ tricarbonyl modes in the excited state indicates an increase in the electron density at the Re-center; hence, the observed ReCl^–^ → ReCl^–^* transition state has LMCT character, opposite to the parent molecule. The lifetime of the ReCl^–^* excited state is very short (20 ps). On an even shorter time scale, the ReCl^–^* ESA bands narrow and shift to higher wavenumbers, typical of vibrational relaxation and/or solvation.^34^

The step from Figure 3b to c, increasing the residence time at the working electrode, is characterized by a strong decrease in the initial ReCl contribution, while the transient signal of ReCl^–^* becomes dominant. The lifetime of ReCl* becomes shorter once a potential is applied, as seen in Figure 3a vs c. We attribute this to the reductive quenching of the excited ReCl* by some of the reduced species, since we also observe a long-lived signal of ReCl^–^ remaining after ReCl* has relaxed to the ground state. A similar behavior has been reported in time-resolved electrochemical fluorescence experiments.^35,36^ The yield of reductive quenching in our case is, however, small, as this residual bleach of ReCl is <10% of the initial bleach. In Figure 3d, the ReCl* and ReCl^–^* signals are not observed any more. Instead, the dominant signals are four negative bands at 1987, 1950, 1883, and 1844 cm^–1^, paired with four positive features with maxima at 2010, 1980, 1912, and 1872 cm^–1^. We assign these signals (yellow traces) to an MLCT-type excited state of the Re–Re dimer.^37^ Complementary to conclusions drawn from 2D-IR spectroscopy,^15^ the equivalence of the lifetimes of all four peaks (2–3 ns) indicates that they originate from one and the same species, which hence must be a hexacarbonyl Re–Re dimer with four observable CO modes,^30^ and not a mixture of two species.

Finally, the transient spectrum in Figure 3e, with the longest residence time at the working electrode, shows two sets of bands. Two new longer-lived bleaches can be seen at 1944 and 1830 cm^–1^. The corresponding positive features are red-shifted. We assign these signals to the doubly reduced Re complex (Re(bpy)), again with an excited state of LMCT character. The shorter-lived sets of bands in Figure 3e, with well separated bleaches at 1995 and 1870 cm^–1^ and positive features at 1983 and 1805 cm^–1^, are assigned to the doubly reduced solvato species Re(bpy)(CO)_3_(acn)^−^ by comparison of the frequencies of the bleach with the literature.^5^

For the other systems with ReCl or ReBr in TBAPF_6_ as the electrolyte, the results are different in terms of the relative amount of reduced species that can be prepared in the interaction region, in accordance with the trend in the CV-SEC measurements (Figure 2). For example, Figure 4a–c shows the gradual appearance of new transient signals in the ReCl/TBAPF_6_ system with a decreasing flow speed. Thanks to their characteristic lifetime of 3 ns, the four (Re–Re dimer) bleach signals (yellow bar) are distinguishable in Figure 4c, while the signals of the parent ReCl and ReCl* are still the most prominent contributions. Without excess chloride, ReCl, ReCl^–^, and the Re–Re dimer are present simultaneously in the interaction region, because ReCl^–^ cannot be accumulated in the TBAPF_6_ electrolyte to same extent as in TBACl, as already discussed in the literature.^5^

For the ReBr/TBAPF_6_ system shown in Figure 5, the transient spectra are even more congested, as already expected from SEC (Figure 2). The decay of ReBr without an applied potential has a lifetime of 38 ns; see Figure 6a. The lifetime of ReBr* becomes shorter once a potential is applied and decreases from 38 ns to less than 30 ns (blue). The signal of ReBr^–^* can hardly be seen. When the Br^–^ ligand is lost, follow-up reactions, such as solvent coordination and dimerization, can occur. Thus, a complex mixture of species is formed with three distinct lifetimes, best seen in the lifetime density map in the right panel of Figure 5.

Discrimination between the Re–Re dimer and Re^–^ can be difficult based on a spectroelectrochemical analysis alone. Both Re–Re and Re^–^ have similar excited-state lifetimes of 2–3 ns; hence, lifetime analysis does not help to distinguish them. They differ with respect to the shift of the ESA bands relative to their GSB signals. However, we have additional control parameters for better discrimination; for example, the applied potential. In Figure 6, we compare results for −1.75 V to −1.80 V with otherwise identical conditions. In Figure 6b, at −1.75 V, the Re–Re dimer is the dominant species, switching to singly reduced Re^–^ at −1.8 V in Figure 6c. We attribute the additional short-lived signals (gray) to the singly reduced dimer Re–Re^–^, based on the bleach positions that are comparable with reported FTIR bands for this molecule in THF^30^; however, it could also be due to a mixture containing solvent-coordinated Re(acn)^−^.

As a last example, we present another useful handle in this variant of spectroelectrochemistry, i.e., the excitation wavelength. Some species have quite different (lower energy) optical absorbance bands compared to the parent ReCl molecule. To illustrate this concept, Figure 7 shows the signals of singly reduced Re(bpy^–•^)(CO)_3_Cl excited at 420 nm versus the signal upon excitation at 840 nm. While the lifetime plot is quite congested in the first case, the ReCl^–^* signals with time constants of around 30 ps are singled out at 840 nm.

In addition, there are some clear differences in the early kinetics <10 ps due to electronic relaxation and vibrational cooling, causing a continuous shift of all vibrational modes. A much larger amount of excess energy has to be dissipated upon 420 nm excitation, as we possibly excite an IL transition located on the bipyridine ligand, which tentatively relaxes to an LMCT state. In the lifetime density maps, the associated spectral shifts can lead to large signals on a <10 ps time scale that should not be mistaken for the presence of a short-lived electrochemical intermediate.

Table 1 summarizes the vibrational frequencies of all compounds encountered in this study, either as observed here or taken from the literature. The excited-state lifetimes are also included.

**Table 1:** Summary of FTIR and trIR Data of Identified Species in the Reduction of Re(bpy)(CO)_3_X Molecules as Obtained from FTIR-SEC (Marked with FTIR) or FullThrOTTLE-trIR (Labelled trIR)

	A’ (1)	A”	A’ (2)	tau (ps)	remarks
ReCl (Cl)/(PF_6_)	2022/2023	1916.3/1916	1899/1900		FTIR
ReBr (PF_6_)	2024	1918	1902		FTIR
ReCl* (Cl)/(PF_6_)	2060	1993^31^	1958^31^	11 ns/29 ns	trIR
ReBr* (PF_6_)				55 ns	trIR
ReCl^–^ (Cl)	1998	1882.9	1866		FTIR
ReCl^–^ (PF_6_)	1999	1884	1868		
ReBr^–^ (PF_6_)	1999	n.a.	n.a.		FTIR

	ν_CO_			tau (ps)	
ReCl^–^*/ReBr^–^*	1989/n.a.	1845/n.a.	1845/n.a.	≈30 ps	trIR
Re(acn)^•^	2011.5	1895(br)^5^			FTIR/ref (5)
Re(acn)^•^*				20 ps	trIR
Re–Re	1985, 1948, 1882, 1851				FTIR
Re–Re*	2014, 1981, 1913, 1871			3 ns	trIR
Re–Re^–^	1998, 1975, 1945, 1870 (br), 1831				trIR
Re–Re^–^*	1986, 1964, 1929, 1910, 1804 (br)			20 ps	trIR
Re^–^	1942.7	1836 (b)			FTIR
Re^–^*	1917	1805	n.a.	2 ns	trIR
Re–Re	1990, 1952, 1986, 1862				ref in THF^30^
Re–Re^–^	1990, 1974, 1951, 1886, 1862, 1837				ref in THF^30^
Re^–^	1945, 1839				ref in THF^30^

## Conclusion

The integration of electrochemistry with time-resolved spectroscopy can provide important new insights. A prerequisite for the FullThrOTTLE-trIR method is the existence of electrochemically semistable intermediates that survive the transport from the working electrode to the interaction region where they are excited. We estimate that this process can take from a few seconds up to a minute at flow rates of 1–50 μL min^–1^ from a channel width and height of 1 mm and 200 μm, respectively, and an average travel distance of 5 mm. This has been illustrated by using simple Re(CO)_3_(bpy)X compounds. Spectral assignment in this case was enormously facilitated by the plethora of available literature on the electrochemistry and spectroscopy of these complexes. This enabled us to optimize the technique and to explore the parameter space, such as potentials and flow rates, which will be used for the investigation of less-known systems in the future.

An important tool is the combination of FullThrOTTLE-trIR data with lifetime density analysis (LDA)^27^, as shown, for example, in Figure 5. A lifetime density map visualizes which signals decay together or change at the same time. Signals with the same decay constants likely belong to the same species. Thus, we can apply this “kinetic sorting” of signals together with the information that we have under which voltage and flow conditions they (gradually) appear for the assignment of absorbance bands to (proposed) reaction intermediates. As long as the excited-state lifetime is shorter than the diffusion limit, it is not influenced by other species in the mixture. However, this may not always be the case and can lead to additional insight into reaction mechanisms. In the present study, many of the observed species have decay constants that are orders of magnitude different from each other. Differentiation is quite easy in these cases. Once decay constants become very similar, as for example for the Re–Re dimer and Re(bpy)(CO)_3_^–^, separation by different parameters (excited-state character, applied potential, and excitation wavelength) was demonstrated.

We found that the excited-state character of the identified reduction intermediates, as well as their excited-state lifetimes, differ greatly. The singly reduced radical anions Re(bpy^•–^)(CO)_3_X and the solvent-coordinated radical Re(bpy^•^)(CO)_3_(acn) exhibited an ESA with ligand-to-metal charge transfer character and excited-state lifetimes in the 20–30 ps range. In contrast, the parent Re(bpy)(CO)_3_X molecule and the Re–Re dimer revealed an MLCT-type (or MLCT/L’LCT) transition with nanosecond lifetimes. The doubly reduced Re complex also has an excited-state lifetime in the lower nanosecond range; however, the CT is of an LMCT type, with increasing electron density at the Re-center upon excitation. There are two major effects at play here: the electron density distribution in the HOMO in each of the intermediates and the energy difference between the HOMO and the LUMO. Re(bpy^•–^)(CO)_3_X and Re(bpy)(CO)_3_^–^ are two species where the electron density at the bipyridine ligand is high; however, the lowest electronic absorption is at much lower energy in the case of Re(bpy^•–^)(CO)_3_X, hence, the shorter excited-state lifetime.

The technique faces a few important challenges. Future versions of the FullThrOTTLE cell will need to control the flow even better; we expect to achieve that by stabilizing the prepressure. A larger working and counter electrode and a shorter interaction channel should improve the overall throughput and degree of electrolysis. Depending on the viscosity of the solvent, a larger electrode area might even become crucial, even though we have reduced the complete sample in this series of experiments while concentrations were as high as we usually use for trIR measurements. We, however, have the impression that this was an almost ideal example. New measurements of lesser-known systems will tell more about limitations also when it comes to potential stability and accuracy or sample homogeneity in the interaction region. It is possible to measure a CV in the cell before starting electrolysis, and the reference electrode is always in contact with fresh sample, so its potential should be reasonably stable in the current spacer configuration.

The FullThrOTTLE-trIR method cannot be applied easily without previous electrochemical and spectroscopic knowledge of the investigated system. However, this applies to spectroelectrochemistry, in general. Even in classic SEC, one would record both the initial spectrum and perform a standard CV measurement before starting any SEC-CV experiment.

To conclude, the FullThrOTTLE-trIR method will enable us to selectively target photocatalytic events in later steps in the catalytic cycle. For example, by knowing the lifetimes of secondary photoactive reaction intermediates, we can better quantify their contribution to the photochemical cycle. This approach is instrumental for understanding the intricate dynamics involved in photochemical reactions, offering insights into reaction mechanisms and providing valuable information for optimizing photocatalytic systems.

## Data Availability

Raw data have been deposited in Zenodo (10.5281/zenodo.13629075).

## Supporting Information Available

Detailed description and drawings of the FullThrOTTLE cell parts (PDF)
