# Supplementary material for: FullThrOTTLE-TrIR: Time-Resolved IR Spectroscopy of Electrochemically Generated Species Using a Full Throughput Optically Transparent Thin-Layer Electrochemical Cell
Source: J Phys Chem C Nanomater Interfaces. 2024 Sep 18;128(38):16040–9. doi: 10.1021/acs.jpcc.4c04947 (PMC11440584; doi:10.1021/acs.jpcc.4c04947)
Supplement: Supplementary file 1 — jp4c04947_si_001.pdf [file jp4c04947_si_001.pdf]

Supporting Information

FullThrOTTLE-TrIR: Time-Resolved IR Spectroscopy of Electrochemically Generated Species Using a Full Throughput Optically Transparent Thin-Layer Electrochemical Cell

Kerstin T. Oppelt* and Peter Hamm

The FullThrOTTLE cell is based on a commercial SPECAC Omnicell for FTIR measurements in solution and a commercial OTTLE cell inset as obtained from Františ̌ek Hartl (University of Reading). The first version of this construction was published in 1991.[1]

We used the back plate and the easy release nuts as well as the back rubber seal from the Omnicell to build the rest of the setup.

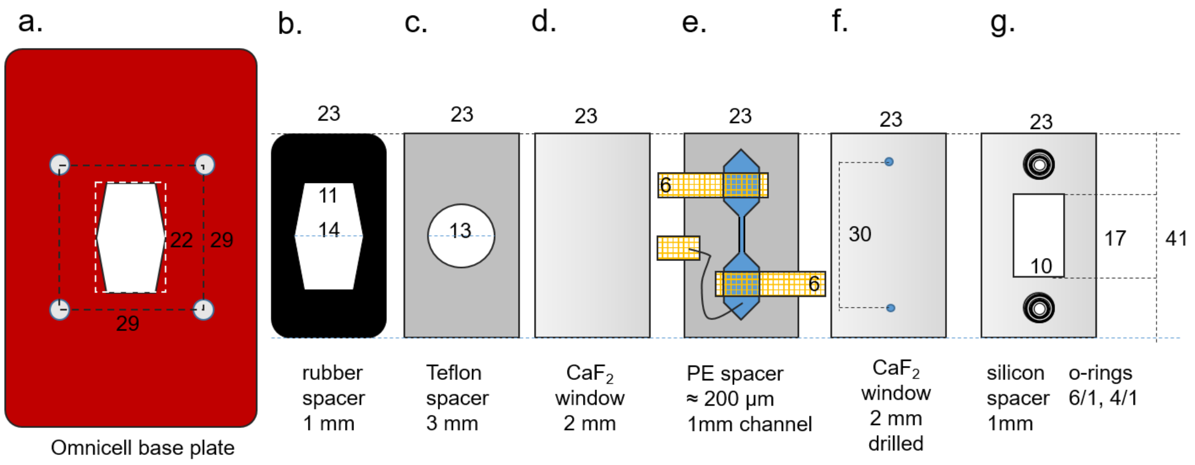

Figure S1 Parts for the FullThrOTTLE cell assembly (back to front) excluding the PEEK gasket for the electrode connections and the top plate with the micro-valves. Measurements in millimeter (mm). a. Omnicell back plate. b. Rubber seal. c. 3 mm thick Teflon spacer with a 13 mm diameter hole. d. 2 mm thick calcium fluoride window. e. Melt- sealed PE spacer with Pt grid and wire electrodes. f. Drilled CaF_2 window (2mm thickness); g. Silicon spacer (1 mm thick) with 6 mm holes holding the 6 & 4 mm O-rings in place.

The melt sealed PE electrode spacer of the traditional OTTLE cell was replaced by a home built one with a flow channel as seen in (Fig.S1e). A 1 mm wide flow channel is cut after the melt assembly of the electrodes, which are long enough (22-25 mm) to be melt sealed at both sides of the 8 mm wide electrode channel. Electrodes are made entirely of Pt grid with a width of 6 mm (GoodFellow, Pt. 99.9%, 0.12 mm opening, wire diameter 0.04 mm, 56% open area) and Pt wire (GoodFellow, 0.04 mm diameter, 99.99% Pt) in between 2 pre-cut PE foils with initially 100 µm thickness each.

The FullThrOTTLE cell is constructed with rectangular CaF_2 windows (41x 23 mm, thickness 2 mm, fabricated by Crystan, UK) separated by the PE spacer with the melt sealed electrodes. One of the two CaF_2 windows has two 1.5 mm diameter holes for the in- and outlet in a distance (center-center) of 30 mm, which matches the outlet of the 2 magnetic valves in the custom top plate.

A PEEK frame that holds the electrode spacer in place containing threaded soldering connectors at the top for the electrodes and has just enough space to allow for the stacking of the actual cell assembly in between is, to the best of our knowledge, commercially available. It has been designed by Františ̌ek Hartl.[3] The frame holding the custom spacer and including

the banana plug connectors is based on this commercial OTTLE cell (Fig. S2). The custom FullThrOTTLE PE spacer is mounted the same way as in a classic OTTLE cell however with some cut-outs to accommodate for the 4 long assembly screws of the Omnicell. This is illustrated in Fig. S2 as well.

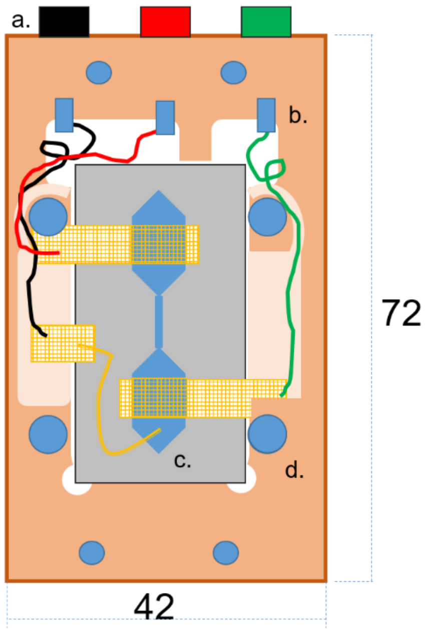

Figure S2 PEEK gasket (8 mm outer thickness) that is used to mount the spacer to the electrode connectors: a. Threaded mini-banana plug connectors. b. Pins of banana plug connector with soldered Cu-wire. c. PE spacer with Pt electrodes. d. PEEK gasket machined to hold the PE spacer in place. This part is based on the commercial OTTLE cell from the University of Reading (Prof. Frantiček Hartl).

The top plate of the commercial Omnicell was replaced by a home-built lid machined from aluminum. Two microvalves are attached which are driven by compatible solenoids using a pulse generator and an electronic driver circuit previously published.[2] The micro-valves (SMLD 300G H J0.1 T1 M F M6 x 0.75, Fritz Gyger AG, Switzerland) have threaded ends that were used to connect them to the top plate.

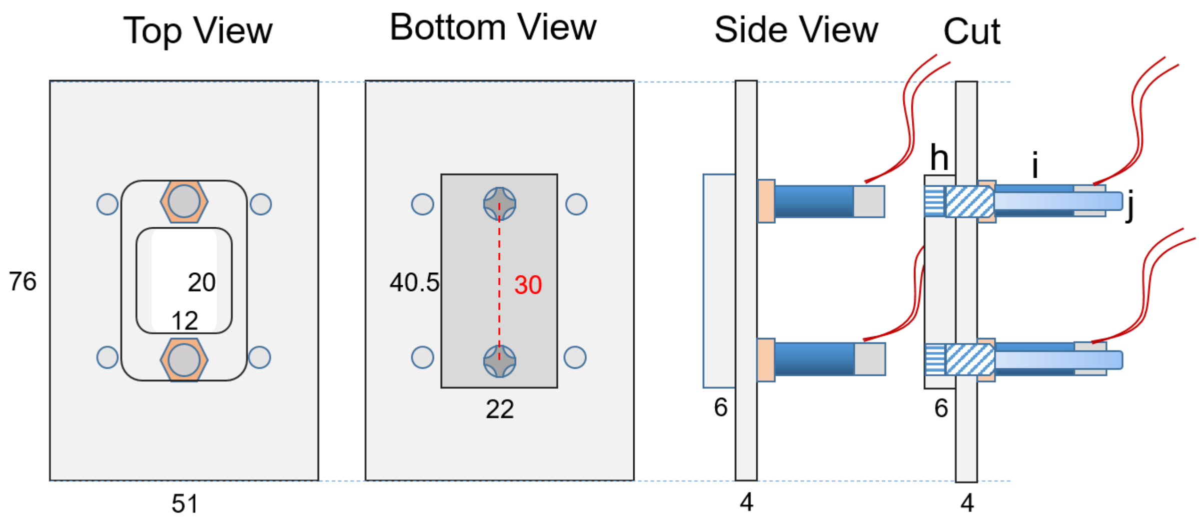

Figure S3 Layout of the top-plate containing the threaded micro-valves. Measurements are in millimeter mm. h. aluminum lid i. Solenoid for micro-valves, j. Micro-valves SMLD 300G H J0.1 T1 M F M6x0.75, Fritz Gyger AG, Switzerland.

The valves have a plane surface at their outlet. This is pressed onto the drilled CaF_2 window with 2 O-rings (6 and 4 mm outer diameter, 1 mm thickness) in beween a 1 mm Silicon spacer. The spacer is necessary to keep the O-rings in place, thus sealing the connection, see Fig. S1g.

The complete assembly (without the PEEK gasket) can be seen in the cut view in Fig.S4.

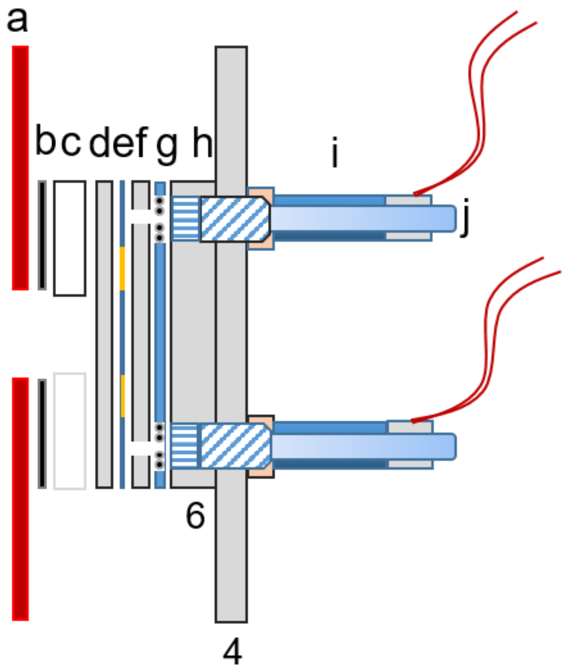

Figure S4 Vertical cut of assembled cell: a. Omnicell back plate. b. Rubber seal. c. 3 mm thick Teflon spacer with a 13 mm diameter hole. d. 2 mm thick calcium fluoride window. e. Melt- sealed PE spacer with Pt grid and wire electrodes. f. Drilled CaF_2 window (2mm thickness); g. Silicon spacer (1mm thickness) with O-rings. h. aluminum lid; i. Solenoid for micro-valves, j. Micro-valves SMLD 300G H J0.1 T1 M F M6x0.75, Fritz Gyger AG, Switzerland.

References:

(1) Krejčik, M.; Daněk, M.; Hartl, F. Simple Construction of an Infrared Optically Transparent Thin-Layer Electrochemical Cell. *J. Electroanal. Chem. Interfacial Electrochem.* **1991**, *317* (1–2), 179–187. https://doi.org/10.1016/0022-0728(91)85012-E.

(2) Buhrke, D.; Ruf, J.; Heckmeier, P.; Hamm, P. A Stop-Flow Sample Delivery System for Transient Spectroscopy. *Rev. Sci. Inst.* **2021**, *92* (12), 123001. https://doi.org/10.1063/5.0068227.

(3) *University of Reading Web Page*. https://research.reading.ac.uk/spectroelectrochemistry /optically-transparent-thin-layer-electrochemical-cells/room-temperature-ottle-cell/ (accessed 2024-09-02)
